# Pharmacological Analysis of GABA_A_ Receptor and Sigma1R Chaperone Interaction: Research Report I―Investigation of the Anxiolytic, Anticonvulsant and Hypnotic Effects of Allosteric GABA_A_ Receptors’ Ligands

**DOI:** 10.3390/ijms24119580

**Published:** 2023-05-31

**Authors:** Mikhail V. Voronin, Stanislav V. Shangin, Svetlana A. Litvinova, Elena V. Abramova, Rustam D. Kurbanov, Inna V. Rybina, Yulia V. Vakhitova, Sergei B. Seredenin

**Affiliations:** Department of Pharmacogenetics, Federal State Budgetary Institution “Research Zakusov Institute of Pharmacology”, Baltiyskaya Street 8, 125315 Moscow, Russia; dnamed@mail.ru (M.V.V.); stas19982010@gmail.com (S.V.S.); sa_litvinova@mail.ru (S.A.L.); ryaskinv@mail.ru (E.V.A.); ramdacato@yandex.ru (R.D.K.);

**Keywords:** GABA_A_ receptors, benzodiazepines, barbiturates, Sigma1R chaperone, elevated plus maze, pentylenetetrazole-induced seizures, sleep duration, BD-1047, NE-100, PRE-084

## Abstract

Two groups of facts have been established in previous drug development studies of the non-benzodiazepine anxiolytic fabomotizole. First, fabomotizole prevents stress-induced decrease in binding ability of the GABA_A_ receptor’s benzodiazepine site. Second, fabomotizole is a Sigma1R chaperone agonist, and exposure to Sigma1R antagonists blocks its anxiolytic effect. To prove our main hypothesis of Sigma1R involvement in GABA_A_ receptor-dependent pharmacological effects, we performed a series of experiments on BALB/c and ICR mice using Sigma1R ligands to study anxiolytic effects of benzodiazepine tranquilizers diazepam (1 mg/kg i.p.) and phenazepam (0.1 mg/kg i.p.) in the elevated plus maze test, the anticonvulsant effects of diazepam (1 mg/kg i.p.) in the pentylenetetrazole-induced seizure model, and the hypnotic effects of pentobarbital (50 mg/kg i.p.). Sigma1R antagonists BD-1047 (1, 10, and 20 mg/kg i.p.), NE-100 (1 and 3 mg/kg i.p.), and Sigma1R agonist PRE-084 (1, 5, and 20 mg/kg i.p.) were used in the experiments. Sigma1R antagonists have been found to attenuate while Sigma1R agonists can enhance GABA_A_Rs-dependent pharmacological effects.

## 1. Introduction

The evidence that the effect of the non-benzodiazepine anxiolytic fabomotizole depends on the interaction with Sigma1R [1] became a key stimulus for setting up the study of GABA_A_ receptor’s (GABA_A_R) interplay with Sigma1R chaperone.

The rationale for the work was also based on previously established neurochemical data, which determined the direction of the search for fabomotizole. GABA_A_Rs are the major mammalian CNS receptors mediating rapid inhibitory neurotransmission. Structurally, they are pentameric ligand-linked chloride channels activated by the endogenous agonist γ-aminobutyric acid (GABA) [2]. In 1994, S. Deutsch et al. summarized the evidence of a decrease in the binding capacity of the benzodiazepine site of GABA_A_Rs upon stress exposure [3]. We confirmed these results. It was found that emotional stress causes a decrease in ^3^H-labeled benzodiazepine binding in open-field (OF) stress-sensitive BALB/c mice [4]. In OF stress-resistant C57Bl/6 mice, no changes were registered [5,6,7]. More potent predator exposure stress caused a decrease in specific benzodiazepine binding in BALB/c and C57Bl/6 mice as well [7,8]. Similar data were obtained for Maudsley reactive (MR) and Maudsley nonreactive (MNRA) rats [9].

Keeping in mind that anxiolytic effect depends on the binding capacity of the GABA_A_R allosteric benzodiazepine site, we focused on the repeatedly confirmed phenomenon of stress-induced specific benzodiazepine binding decrease in the search for a new anxiolytic [10,11]. In other words, we believed that a substance preventing the stress-induced drop in specific benzodiazepine binding would possess anxiolytic effects.

Screening studies allowed us to select the 5-ethoxy-2-[2-(morpholino)-ethylthio]benzimidazole dihydrochloride compound (fabomotizole), which prevented stress-induced decrease in specific benzodiazepine binding [8,12]. Anxiolytic effect of fabomotizole in standard tests was exhibited in a wide range of doses [5,8]. It did not cause sedation, myorelaxation, or other undesirable effects of benzodiazepines, which was confirmed both in the experiment and clinically [5,13,14]. Fabomotizole was registered in Russia in 2006 as anxiolytic Afobazole.

Radioligand studies of fabomotizole interaction with the GABA_A_Rs’ benzodiazepine site revealed no ligand properties [15]. To elucidate the primary interaction of fabomotizole, we performed radioligand assays on a number of targets that theoretically mediate the anxiolytic effect (Eurofins Scientific). Fabomotizole was found to interact with chaperone Sigma1R (K_i_ = 5.9 μM), regulatory sites of NRH: quinone reductase 2 (NQO2, MT_3_ receptor K_i_ = 0.97 μM) and monoamine oxidase A (MAO-A K_i_ = 3.6 μM), and MT_1_ receptor (K_i_ = 16 μM) [15]. Among the metabolites of fabomotizole, compound 2-[2-(3-oxomorpholin-4-il)-ethylthio]-5-ethoxybenzimidazole hydrochloride (M-11) was identified as the main one [16]. M-11 has ligand properties only to the MT_3_ receptor, and its anxiolytic effect was significantly weaker than that of fabomotizole [17].

Thus, the ability of fabomotizole to prevent stress-induced drop in specific benzodiazepine binding, and its interaction with chaperone Sigma1R, defining the anxiolytic properties, were established. These results determined the task of investigating the dependence of drugs effects mediated through GABA_A_Rs on the interaction of GABA_A_Rs with Sigma1R.

In the present work, using Sigma1R antagonists and agonists, we studied anxiolytic, anticonvulsant, and hypnotic effects.

## 2. Results

### 2.1. Anxiolytic Effect of Benzodiazepines

**Figure 1:**
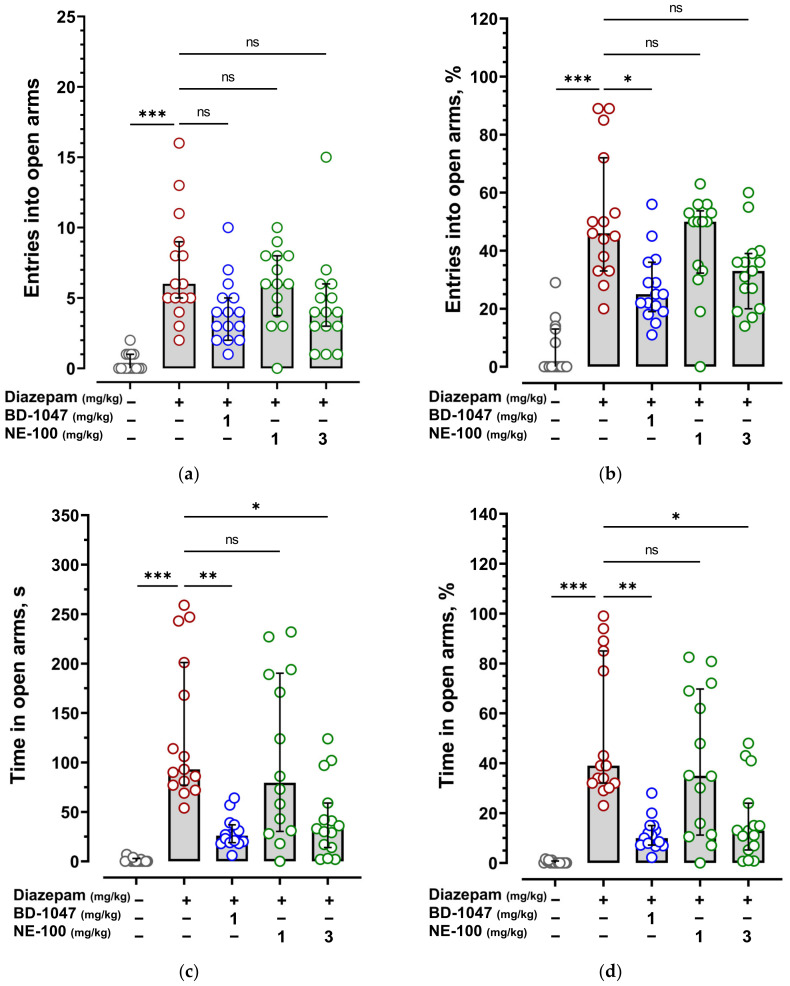
Influence of Sigma1R antagonists BD-1047 and NE-100 on the anxiolytic effect of diazepam in the elevated plus maze test. (**a**) The number of entries into the open arm (N open); (**b**) percentage of the open-arm entries (%N open); (**c**) time in seconds spent in the open arm (T open); (**d**) percentage of time spent in the open arm (%T open). Vehicle 2 and diazepam (1.0 mg/kg) were injected i.p. 30 min prior to the EPM exposition. Vehicle 1, selective Sigma1R antagonists BD-1047 (1.0 mg/kg), and NE-100 (1.0 and 3.0 mg/kg) were injected i.p. 30 min prior to diazepam. Data are presented as median with interquartile range. Statistically significant differences according to the Kruskal–Wallis test and Dunn’s multiple comparison test: ns—not significant; * *p* < 0.05; ** *p* < 0.01; *** *p* < 0.001.

**Figure 2:**
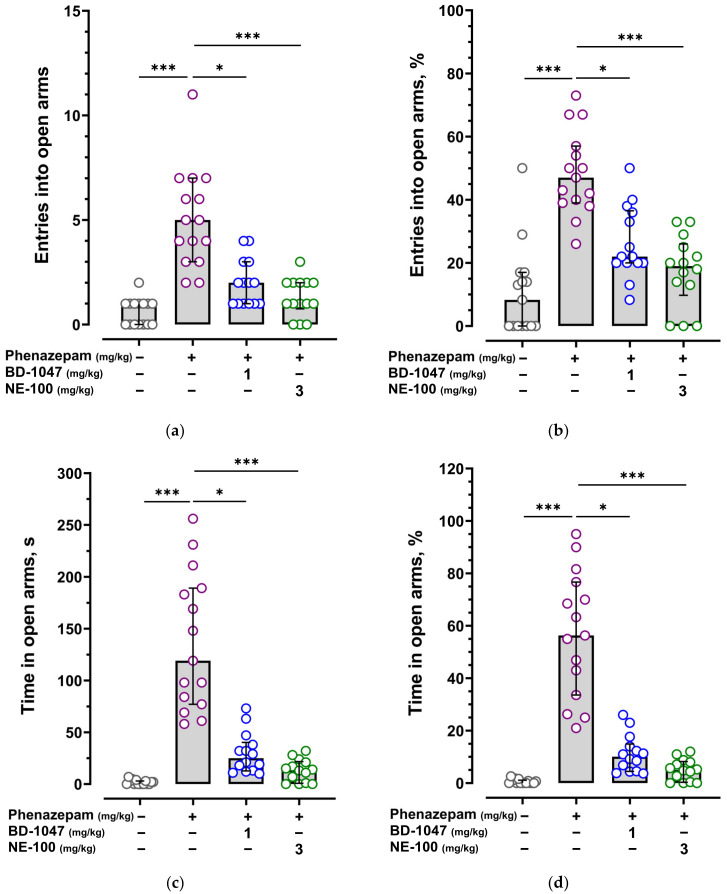
Influence of Sigma1R antagonists BD-1047 and NE-100 on the anxiolytic effect of phenazepam in the elevated plus maze test. (**a**) The number of entries into the open arm (N open); (**b**) percentage of the open-arm entries (%N open); (**c**) time in seconds spent in the open arm (T open); (**d**) percentage of time spent in the open arm (%T open). Vehicle 2 and phenazepam (0.1 mg/kg) were injected i.p. 30 min prior to the EPM exposition. Vehicle 1, selective Sigma1R antagonists BD-1047 (1.0 mg/kg), and NE-100 (3.0 mg/kg) were injected i.p. 30 min prior to phenazepam. Data are presented as median with interquartile range. Statistically significant differences according to the Kruskal–Wallis test and Dunn’s multiple comparison test: * *p* < 0.05; *** *p* < 0.001.

Anxiolytic effects of diazepam and phenazepam were reproduced on BALB/c mice in the elevated plus maze test (EPM). Diazepam at a dose of 1.0 mg/kg and phenazepam at a dose of 0.1 mg/kg administered 30 min before the animals’ exposure to the EPM statistically significantly increased number of entries and time spent in the open arms (Figure 1 and Figure 2, Tables S2 and S3).

The Sigma1R antagonist BD-1047 at a dose of 1.0 mg/kg injected 30 min before diazepam interfered with the anxiolytic effect of the drug by reducing the % number of entries into the open arms (%N open *p* < 0.05) and time spent in the open arms (T open *p* < 0.01; %T open *p* < 0.01) (Figure 1, Table S2). The Sigma1R antagonist NE-100 at a dose of 1.0 mg/kg administered 30 min before diazepam did not influence its anxiolytic effect. However, when the dose was increased to 3.0 mg/kg, NE-100 reduced the time spent in the open arms (T open *p* < 0.05; %T open *p* < 0.05) (Figure 1, Table S2). Administration of BD-1047 prior to diazepam resulted in a peculiar feature of increased number of entries into closed arms (N closed) compared to NE-100 (*p* < 0.01) (Figure S1, Table S2). Pretreatment with the Sigma1R agonist PRE-084 at a dose of 1.0 mg/kg had no influence on diazepam effect in the EPM (Table S4).

Sigma1R antagonists BD-1047 at a dose of 1.0 mg/kg and NE-100 at a dose of 3.0 mg/kg, administered 30 min before phenazepam, inhibited its anxiolytic effect by reducing the number of entries into the open arms (N open *p* < 0.05, *p* < 0.001; %N open *p* < 0.05, *p* < 0.001) and time spent in the open arms (T open *p* < 0.05, *p* < 0.001; %T open *p* < 0.05, *p* < 0.001) (Figure 2, Table S3), consequently decreasing the total entries parameter (N total *p* < 0.05, *p* < 0.01) (Figure S2, Table S3).

Administration of vehicle before the EPM exposure had no effect on the behavior of BALB/c mice except for a slight increase in the % of time spent in the open arms (%T open; *p* = 0.048) compared with intact animals. The Sigma1R antagonists BD-1047 at a dose of 1.0 mg/kg and NE-100 at doses of 1.0 and 3.0 mg/kg, injected 60 min before testing, had no effect on mouse behavior in the EPM (Table S1).

To exclude the association of Sigma1R antagonists’ effects on the anxiolytic action of diazepam and phenazepam with the affinity to the benzodiazepine site of GABA_A_Rs, the competitive displacement of [N-methyl-^3^H] flunitrazepam in P2 fractions of BALB/c mouse brain homogenates was evaluated. BD-1047 and NE-100 were found to have no affinity to the benzodiazepine site of GABA_A_R (Figure S3).

### 2.2. Anticonvulsant Effect of Benzodiazepines

**Figure 3:**
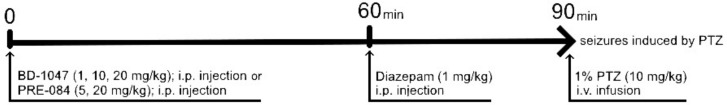
Drug administration design in a model of pentylenetetrazole-induced seizures in mice. The i.p. injection of BD-1047 (1, 10 and 20 mg/kg), PRE-084 (5 and 20 mg/kg), or their vehicle marked the start of the experiment. Diazepam (1 mg/kg i.p.) was administered 60 min after the first injection. A 1% PTZ solution was administered i.v. 90 min after the first injection.

Clonic jerks, generalized clonic seizures, generalized tonic seizures, and their time of onset were recorded during the experiment (Figure 3).

**Figure 4:**
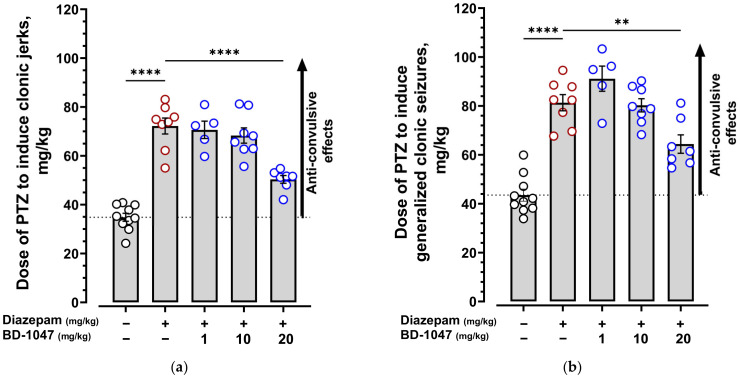

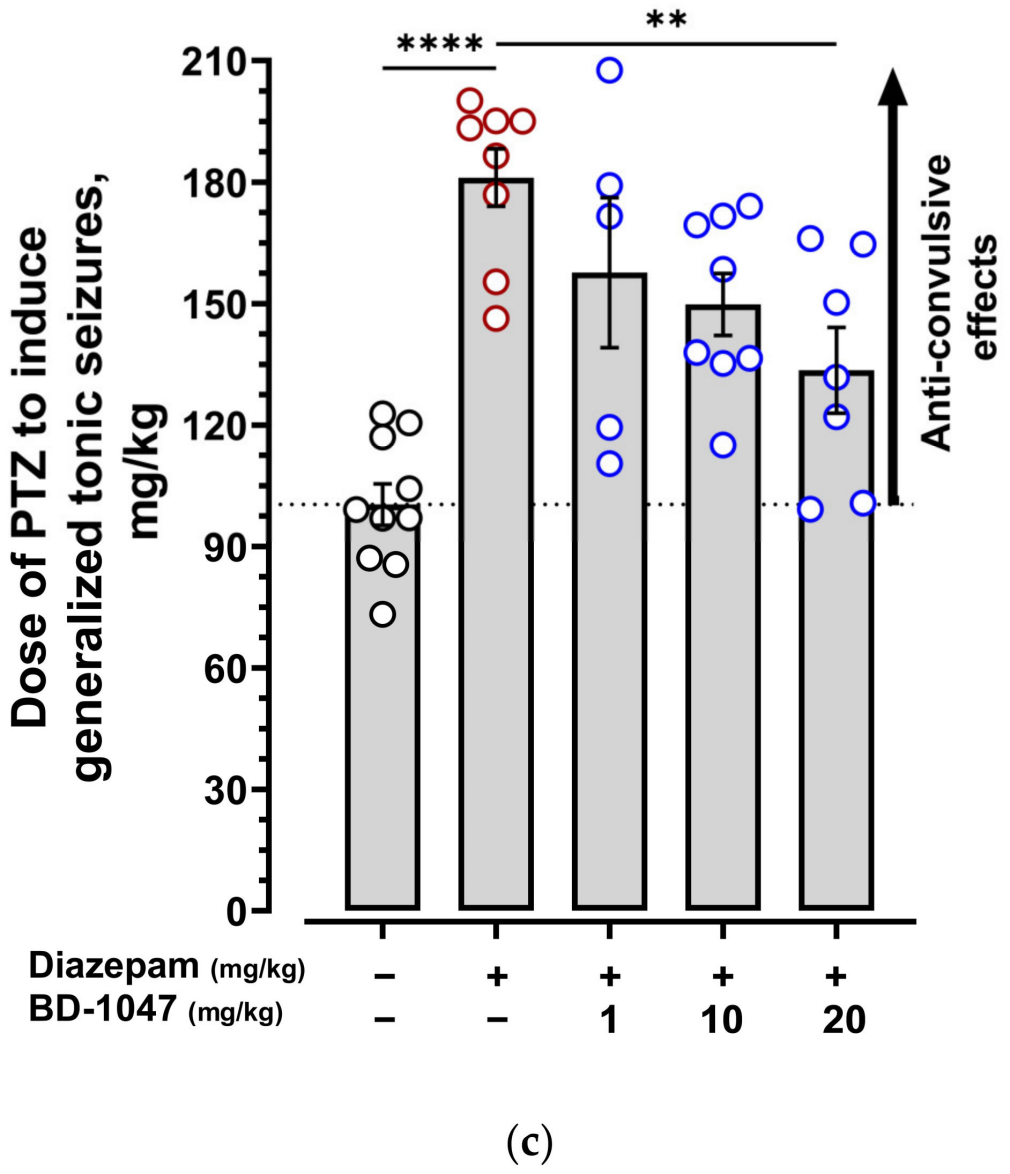
Effect of the selective Sigma1R antagonist BD-1047 on the anticonvulsant activity of diazepam in the pentylenetetrazole-induced seizure model. (**a**) Clonic jerks; (**b**) generalized clonic seizures; (**c**) generalized tonic seizures. The dotted line represents the threshold for PTZ-induced seizures. The upward arrow indicates that the compound increases the seizure threshold and has anticonvulsant effects. BD-1047 (1, 10, and 20 mg/kg) was injected i.p. 90 min before PTZ infusion. Diazepam (1 mg/kg) was administered i.p. 30 min before the PTZ infusion. Data are presented as mean ± S.E.M. Statistically significant differences vs. diazepam (1 mg/kg) according to one-way ANOVA with Dunnett post-hoc test: ** *p* < 0.01; **** *p* < 0.0001.

**Figure 5:**
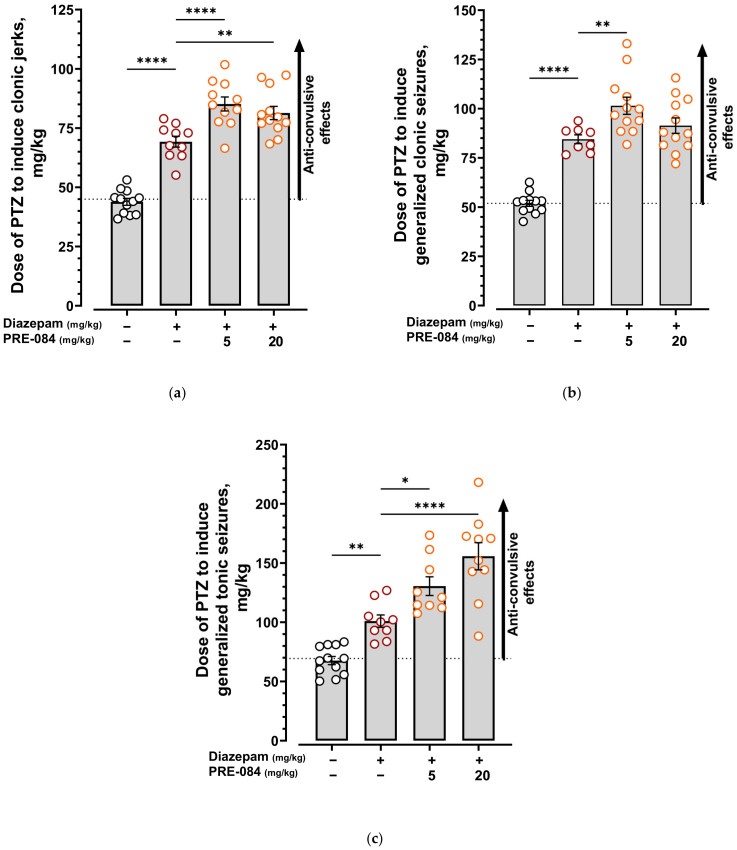
Effect of the selective Sigma1R agonist PRE-084 on the anticonvulsant activity of diazepam in the pentylenetetrazole-induced seizure model. (**a**) Clonic jerks; (**b**) generalized clonic seizures; (**c**) generalized tonic seizures. The dotted line represents the threshold for PTZ-induced seizures. The upward arrow indicates that the compound increases the seizure threshold and has anticonvulsant effects. PRE-084 (5 and 20 mg/kg) was administered i.p. 90 min before the PTZ infusion. Diazepam (1 mg/kg) was administered i.p. 30 min before the PTZ infusion. Data are presented as mean ± S.E.M. Statistically significant differences vs. diazepam (1 mg/kg) according to one-way ANOVA with Dunnett post-hoc test: * *p* < 0.05; ** *p* < 0.01; **** *p* < 0.0001.

Intravenous injections of pentylenetetrazole (PTZ) to control groups of mice induced clonic jerks and generalized clonic seizures at doses ranging from 35 to 52 mg/kg and generalized tonic seizures at doses of 68 and 100 mg/kg (Figure 4 and Figure 5, Tables S5 and S6). In control experiments, BD-1047 at doses of 1, 10, and 20 mg/kg had no effect on PTZ-induced seizure thresholds (Figure 4 and Figure S4, Table S5). Diazepam administered at a 1 mg/kg dose 30 min before PTZ increased the clonic jerk threshold 2.9-fold and generalized clonic and tonic seizures 1.8-fold. Pre-injected BD-1047 (1–20 mg/kg) attenuated the effect of diazepam in a dose-dependent manner, statistically significantly reducing the PTZ dose inducing generalized clonic and tonic seizures by 21% and 26% at the 20 mg/kg dose (Figure 4, Table S5). The findings indicate the ability of a 20 mg/kg BD-1047 dose to inhibit the anticonvulsant effect of diazepam.

PRE-084 showed no independent effect on PTZ-induced seizure activity (Figure S5, Table S6). Pretreatment of mice with 5 mg/kg PRE-084 statistically significantly enhanced the anticonvulsant effect of diazepam at a 1 mg/kg dose, increasing all seizure reaction thresholds (Figure 5, Table S6). Raising the dose of PRE-084 to 20 mg/kg increased the anticonvulsant effect of diazepam only on the thresholds of clonic jerks and generalized tonic seizures (Figure 5a,c, Table S6) but not generalized clonic seizures (Figure 5b, Table S6). PRE-084 increased the anticonvulsant effect of diazepam in a dose-dependent manner when registering the threshold of generalized tonic seizures (Figure 5c, Table S6). The obtained data indicate the ability of high doses of Sigma 1R agonist PRE-084 to enhance the anticonvulsant effect of diazepam.

### 2.3. Hypnotic Effect of Pentobarbital

**Figure 6:**
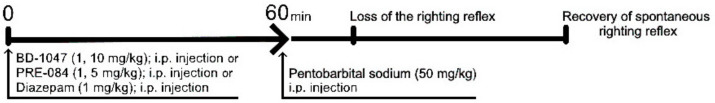
Drug administration design in a pentobarbital-induced sleep model in mice. The i.p. administration of BD-1047 (1 and 10 mg/kg), PRE-084 (1 and 5 mg/kg), diazepam (1 mg/kg), or their vehicles marked the start of the experiment. Pentobarbital was injected i.p. at a dose of 50 mg/kg 60 min after the first injection. After pentobarbital administration, the time to fall asleep and the duration of sleep were recorded. The time to fall asleep was registered by the loss of the righting reflex, and sleep duration was recorded from the moment of falling asleep to the moment of spontaneous righting reflex recovery.

To evaluate the influence of Sigma1R ligands on the pharmacological effects dependent on the barbiturate binding site of GABA_A_Rs, an experiment was performed on a pentobarbital-induced sleep model in mice (Figure 6).

**Figure 7:**
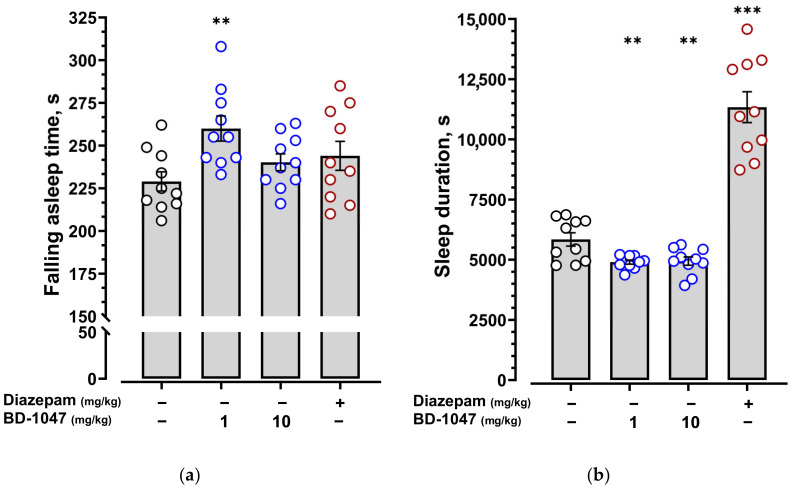
Effect of the selective Sigma 1R antagonist BD-1047 on pentobarbital-induced sleep. (**a**) Falling asleep time; (**b**) sleep duration. BD-1047 (1 and 10 mg/kg) was administered i.p. 60 min before pentobarbital administration. Pentobarbital was administered i.p. at a dose of 50 mg/kg. Data are presented as mean ± S.E.M. Statistically significant differences vs. control group according to one-way ANOVA with Dunnett post-hoc test: ** *p* < 0.01; *** *p* < 0.001.

BD-1047 at a dose of 1 mg/kg prevented the hypnotic effect of pentobarbital by statistically significantly increasing the falling asleep time (Figure 7a, Table S7) and reducing pentobarbital-induced sleep duration (Figure 7b, Table S7). At a dose of 10 mg/kg, BD-1047 statistically significantly reduced only pentobarbital-induced sleep duration (Figure 7b, Table S7).

**Figure 8:**
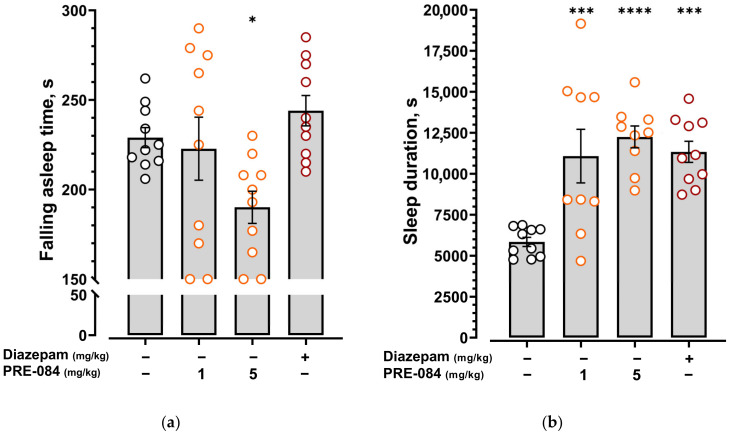
Effect of the selective Sigma 1R agonist PRE-084 on pentobarbital-induced sleep. (**a**) Falling asleep time; (**b**) sleep duration. PRE-084 (1 and 5 mg/kg) was administered i.p. 60 min before pentobarbital administration. Pentobarbital was administered i.p. at a dose of 50 mg/kg. Data are presented as mean ± S.E.M. Statistically significant differences vs. control group according to one-way ANOVA with Dunnett post-hoc test: * *p* < 0.05; *** *p* < 0.001; **** *p* < 0.0001.

The Sigma1R agonist PRE-084 at a dose of 1 mg/kg enhanced the effects of pentobarbital by statistically significantly increasing pentobarbital-induced sleep duration (Figure 8b) but not changing the falling asleep time (Figure 8a, Table S7). Increasing the dose of PRE-084 to 5 mg/kg revealed a statistically significant decrease in falling asleep time (Figure 8a, Table S7) and an increase in pentobarbital-induced sleep duration (Figure 8b).

The results indicate opposite effects of Sigma1R antagonists and agonists on the hypnotic properties of pentobarbital.

## 3. Discussion

GABA_A_R is a heteropentameric ligand-gated ion channel of the Cys-loop family, responsible for incoming Cl^−^ current and subsequent inhibition of neuronal excitability through hyperpolarization and decreased membrane resistivity [2,18]. To date, nineteen subunits of GABA_A_Rs have been described: α1–6, β1–3, γ1–3, δ, ε, θ, π, and ρ1–3. The GABA_A_R α1β2γ2 subtype is the most common in the CNS (∼60%). Less common are α2β3γ2 (∼15–20%) and α3βnγ2 (∼10–15%). Receptors containing α4-, α5-, and α6- subunits, as well as β1-, γ1–3, δ-, π-, and θ-subunits, form a minor population [2,19]. Depending on their subunit composition, cellular localization, and expression in brain structures, GABA_A_Rs have different biophysical properties and allosteric binding sites formed by subunit interfaces, which determine the assignment of GABA_A_Rs to a particular subtype [20,21,22,23]. The ligands of these sites induce conformational changes in the corresponding subunits and modulate the activity of GABA_A_Rs, affecting the channel opening both positively (barbiturates, benzodiazepines, ethanol, etomidate, glutethimide, anesthetics, and some neurosteroids with a reduced 3α-hydroxy A-ring) and negatively (pregnenolone sulfate and Zn^2+^). Several positive allosteric modulators (PAMs) of GABA_A_Rs are widely used to treat anxiety, insomnia, and seizures including status epilepticus. For a review, see [2,11,19,24,25,26].

PTZ was shown to act as a GABA_A_R competitive antagonist through the binding with the picrotoxin site, decreasing the frequency of Cl^−^ channel opening rather than an open-channel state duration [27,28]. We tested the effect of selective Sigma1R agonist PRE-084 and antagonist BD-1047 in a mouse PTZ-induced seizure model to prove that Sigma1R is engaged in the regulation of anti-convulsive activity of diazepam. Single administration of PTZ at a submaximal dose is generally used to model epileptic seizures in rodents, which is believed to reflect myoclonic seizures in humans [29].

The main full-length isoform of the mammalian Sigma1R chaperone is constituted by 223 amino acid residues, which form the transmembrane and chaperone domains, as well as the ligand-binding site [30,31,32,33,34]. The amino acid sequence of mouse Sigma1R is 90% identical to that of humans [35,36]. In the CNS, Sigma1R is expressed in both neurons and glial cells [37], where it is predominantly localized in mitochondria-associated membranes (MAMs) of endoplasmic reticulum (ER) [34,38,39,40,41,42]. Sigma1R is capable of interacting with ER and cytoplasmic membrane client proteins and regulating their functional activity [43,44,45,46].

In the present study, the GABA_A_R-dependent anxiolytic and anticonvulsant effects of benzodiazepines (diazepam, phenazepam) and the hypnotic effect of pentobarbital were attenuated by Sigma1R ligands with antagonistic activity.

The negative influence of Sigma1R antagonists on the anxiolytic-like effect of benzodiazepines is consistent with the anxiety-like behavior of *Sigmar1^−/−^* mice in standard tests [47]. In our study, the Sigma1R agonist PRE-084 (1 mg/kg) did not alter the anxiolytic-like effect of diazepam, but in in vivo experiments, compounds with Sigma1R agonist properties can interfere with anxiety-like behavior [48,49,50,51,52].

The attenuation of diazepam anticonvulsant action by Sigma1R antagonist BD-1047 established in our study is consistent with the results of E. Vavers et al. [53], who showed a decrease in the threshold of seizures induced by PTZ [28] and GABA_A_Rs antagonist bicuculine [54] in mice with *Sigmar1* gene inactivation. These effects of PTZ and bicuculine were independent of the expression levels of α4, α5, β3, γ2, and δ GABA_A_R subunits, which did not change in *Sigmar1^−/−^* mice [53,55]. Similar to [56], our study showed that Sigma1R antagonists had no effect on the pro-convulsive activity of PTZ at doses less than 20 mg/kg. It is important to note that the independent pro-convulsive activity of NE-100 detected at doses above 25 mg/kg was attenuated by *Sigmar1* gene inactivation [53,56]. The effects of Sigma1R antagonists on GABA_A_R-dependent effects were recorded not only in vivo but also in vitro. Thus, NE-100 increased synaptosomal transporter-mediated [^3^H]-GABA uptake but enhanced the negative effect of flumazenil, a benzodiazepine GABA_A_R site antagonist, in the process. Moreover, NE-100 reduced stimulated transporter-mediated and exocytotic release of [^3^H]-GABA from nerve terminals [57]. The above data together with the lack of affinity of BD-1047 and NE-100 to the benzodiazepine site of GABA_A_Rs and competition of NE-100 for [^3^H]-muscimol binding site [53] indicate the ability of Sigma1R antagonists to prevent pharmacological effects of PAMs of GABA_A_Rs by changing functional activity of Sigma1R at relatively low doses.

In our study, PRE-084 at doses of 5 and 20 mg/kg increased the anticonvulsant effect of diazepam. It also shortened the falling asleep time (5 mg/kg PRE-084) and prolonged pentobarbital-induced sleep duration (1 and 5 mg/kg PRE-084). Similarly, prior single administration of the non-selective Sigma1R ligand opipramol, which has anxiolytic properties [58], also increased the latency of the PTZ-induced clonic seizures [59]. The data on the attenuation of seizures by Sigma1R ligands with agonist properties [60] are consistent with the results obtained in the anhedonia modeling with the proconvulsive GABA_A_R antagonist picrotoxin. Only fluvoxamine and S-(+)-fluoxetine, high-affinity Sigma1R ligands with agonist properties [61,62], were shown to attenuate picrotoxin-induced anhedonia [63], unlike antidepressants with lower affinity for Sigma1R [64]. Pre-administration of Sigma1R antagonist BD-1047 prevented this effect of fluvoxamine. However, the anti-anhedonic effect of paroxetine interacting with Sigma1R in the micromolar concentration range was manifested in this experimental model only when administered together with the selective Sigma1R agonist (+)-SKF-10.047 [64]. In PTZ-induced seizures and kainic-acid-induced status epilepticus models, compounds with agonist activity to Sigma1R (SKF-10.047, dextromethorphan, and carbetapentane) and Sigma1R positive modulators (SKF83959, SOMCL-668, and E1R) showed anticonvulsant properties. The effects of Sigma1R positive modulators were eliminated by prior administration of BD-1047 or NE-100 [56,65,66]. The opposite influence of Sigma1R antagonists and agonists on the effects of PAMs of GABA_A_R found in our work is consistent with the study results where PRE-084 attenuated NE-100-induced seizures in mice [56]. The effects of Sigma1R ligands and modulators in various seizure models are discussed in the review [67]. Thus, most experimental data point to attenuation of seizures by compounds with Sigma1R agonist activity. However, in Zebrafish, the *scn1a* mutant model of Dravet syndrome antagonistic ligand regulation of Sigma1R attenuates epileptiform behavior [68]. Seizure disorders in Dravet syndrome mouse models caused by mutations in the *Scn1a* gene are accompanied by disturbances in GABAergic firing in hippocampal neurons [69], which may also indicate attenuation of epileptiform behavior due to Sigma1R-dependent modulation of GABA_A_Rs activity.

In our study, the Sigma1R antagonist BD-1047 attenuated the hypnotic effect of pentobarbital. The current literature does not provide sufficient data on the relationship of Sigma1R with sleep regulation and the effects of barbiturates. However, orexin-system-mediated/controlled sleep–wake cycle effects may be dependent on Sigma1R [70,71]. The role of chaperone-dependent reduction of ER stress in sleep normalization is also known [72].

Based on the data on the relationship between the subunit composition of GABA_A_Rs and the effects mediated by them, we might predict which subtypes of GABA_A_Rs could be involved in the regulatory effects of Sigma1R. Diazepam binds with the benzodiazepine site of GABA_A_Rs at the α+/γ2– interfaces. Synaptic subtypes of GABA_A_Rs that mediate the sedative (α1βγ2) [73], anticonvulsant (α1-containing GABA_A_Rs) [74], hypnotic (α2-, α3-, or α5-containing GABA_A_Rs) [19] and anxiolytic (α2βγ2 as well as α2/α3-containing GABA_A_Rs) [75] effects were shown to be the main targets of diazepam. Pentobarbital predominantly allosterically modulates α4β2γ2, α4β2δ [76], and α1β3δ [77] GABA_A_Rs subtypes. Sigma1R-mediated effects may also depend on the interactions with extrasynaptic GABA_A_Rs subtypes, predominantly represented by α4–6, β2/3, and δ subunits [21,78,79,80].

However, given the opposite influence of Sigma1R antagonists and agonists on the effects of GABA_A_R PAMs interacting with different GABA_A_R binding sites, we cannot rule out other Sigma1R-dependent mechanisms of GABA_A_R regulation modulating the functional activity of the latter in general. Possible mechanisms of Sigma1R chaperone influence on GABA_A_R-dependent effects can be discussed based on the knowledge of Sigma1R physiological properties.

First, Sigma1R-dependent influence on the effects of GABA_A_R PAMs may be mediated by chaperone properties toward cytoplasmic membrane proteins involved in the regulation of anxiety and seizures [44]. Under misfolded proteins’ accumulation conditions (ER stress) or ligand activation, Sigma1R is capable of intracellular translocation within lipid domains, including the plasma membrane region [12,38,81,82]. On the other hand, antagonistic action on the chaperone is able to reduce Sigma1R levels in the cell surface membrane and the expression of α and β subunits of GABA_A_Rs in vivo [83]. Examples of such target proteins of the chaperone are cannabinoid (CB_1_R) [67,84,85,86] and NMDA glutamate (GluN1, GluN2) receptors [87,88,89,90,91], ligands of which inhibit anxiolytic effects of benzodiazepine tranquilizers [92,93]. To date, no data on protein–protein interactions of Sigma1R with GABA_A_Rs have been found in the available literature. Clarification of these issues requires further investigation.

Second, it is possible that the effects of PAMs of GABA_A_Rs are dependent on the dissociation of activated Sigma1R from the main chaperone ER BiP (GRP78, HSPA5) [32,38,46], which contributes to the increased activity of both chaperones [32,38,94,95,96], enhanced protein folding [43,97], and regulation of ER stress sensor IRE1α, which triggers the unfolded protein response (UPR) signaling [39,42,98]. This assumption is supported by data on the interaction of the BiP chaperone with GABA_A_Rs subunits [99,100,101,102] and the enhancement of their folding and cell trafficking properties [103,104,105]. According to [106] the anticonvulsant drug valproate [107,108] is able to enhance BiP expression without activating ER stress.

Third, Sigma1R plays an important role in the maintenance of Ca^2+^ cell homeostasis [38,109,110,111]. Interestingly, Sigma1R activation is also accompanied by PKC translocation to the membrane vicinity [112], which may contribute to the phosphorylation of GABA_A_Rs and enhance the potentiating effect of PAMs on GABA_A_Rs [113]. The influence of Sigma1R antagonists on the effects of PAMs of GABA_A_Rs detected in our study is consistent with the down-regulation of protein kinase C (PKC) in *Sigmar1^−/−^* mice [114]. E. Sallard et al. [20] discuss the possibility of activation and inhibition of GABA_A_Rs under conditions of Ca^2+^ release from the ER at low and high concentrations, respectively [115,116]. A similar regulation of GABA_A_Rs may depend on the activated Sigma1R ability to increase intracellular calcium, eliminated by chaperone antagonists [117,118,119]. The contribution of Sigma1R-dependent regulation of L- and N-type voltage-gated Ca^2+^ channels [120] to the effects mediated by GABA_A_Rs [121,122] is not excluded.

Fourth, it is plausible that chaperone Sigma1R contribution to the regulation of GABA_A_R-dependent effects is mediated by Sigma1R action on cell membranes. The influence of Sigma1R on the lipid environment of GABA_A_Rs is consistent with the established role of Sigma1R in cholesterol metabolism in cell membranes and reorganization of lipid raft proteins [42,123,124,125,126,127,128]. This property of Sigma1R can influence the functional activity of GABA_A_R, which depends on the lipid environment [129,130]. This assumption is supported by the fact that the stimulatory effect of GABA and diazepam on GABA_A_R chloride current depends on lipid raft integrity [131]. In turn, pentobarbital anesthesia contributes to disruption of lipid–protein stability [132].

Therefore, our study demonstrates for the first time the opposite action of Sigma1R antagonists and agonists on the effects of allosteric modulators of GABA_A_R. The findings demonstrate that Sigma1R chaperone contributes to the mechanisms of GABA_A_Rs-dependent effects. Further studies will be aimed at revealing the mechanisms of interaction between Sigma1R and GABA_A_Rs.

## 4. Materials and Methods

### 4.1. Chemicals

The following chemicals were used: diazepam (J.S.C. «Organica», Novokuznetsk, Russia), phenazepam (FSBI “Zakusov Institute of Pharmacology”, Moscow, Russia), pentobarbital sodium salt (FSBI “Zakusov Institute of Pharmacology”, Moscow, Russia), pentylenetetrazole (Sigma Aldrich, Burlington, MA, USA), polysorbate-80 (tween-80) (Sigma Aldrich, USA), polyethylene glycol 400 (Sigma-Aldrich, Burlington, MA, USA), BD-1047 hydrobromide (Tocris Bioscience, Bristol, UK), NE-100 hydrochloride (Santa Cruz Biotechnology, Dallas, TX, USA), [N-methyl-^3^H] flunitrazepam (Amersham, UK), BD-1047 hydrobromide (Tocris Bioscience, Bristol, UK), NE-100 hydrochloride (Santa Cruz Biotechnology, Dallas, TX, USA), Tris(hydroxymethyl)aminomethane (Sigma-Aldrich, Burlington, MA, USA), sucrose (Sigma-Aldrich, Burlington, MA, USA), 1,4-dioxane (Ecos-1, Moscow, Russia), naphthalene (Sigma-Aldrich, Burlington, MA, USA), 2,5-diphenyloxazole (Sigma-Aldrich, Burlington, MA, USA), and 1,4-Bis(5-phenyl-2-oxazolyl)benzene (Sigma Aldrich, Burlington, MA, USA).

### 4.2. Experimental Animals

A total of 207 male BALB/c mice (20–22 g) were used for the EPM. BALB/c mice were obtained from Pushchino Breeding Center (Branch of the Institute of Bioorganic Chemistry, Russian Academy of Sciences). Studies in models of pentylenetetetrazole-induced seizures and barbiturate-induced sleep were performed on male ICR mice (25–40 g) obtained from the “Stolbovaya” Nursery of Laboratory Animals at the Scientific Center for Biomedical Technology of the Federal Medical and Biological Agency (Stolbovaya, Moscow Region, Russian Federation). Animals were housed under standard vivarium conditions (20–22  °C, 30–70% humidity, 12 h light/dark cycle) in plastic cages with sawdust bedding and 6–12 animals per cage.

### 4.3. Ethical Approval

All experimental procedures were approved by the bioethics committee of the FSBI “Zakusov Institute of Pharmacology”, protocols #09 of 29 October 2022 and #03 of 31 January 2023. All applicable national [133] and international [134] guidelines for the care and use of experimental animals were followed.

### 4.4. In Vivo Study

#### 4.4.1. In Vivo Experimental Design

In vivo experimental design was developed in compliance with the 3R principles [135]. (1) Elevated plus maze test: For injections, BD-1047 hydrobromide and NE-100 hydrochloride were dissolved in water (vehicle 1), while diazepam and phenazepam were dissolved in 50% PEG 400 solution (vehicle 2) immediately before administration [136,137,138]. Injections were made intraperitoneally (0.1 mL/10 g body weight). Diazepam at a dose of 1.0 mg/kg [8,139,140] and phenazepam at a dose of 0.1 mg/kg [141] were injected 30 min prior to the EPM exposition. Selective Sigma1R antagonists BD-1047 at a dose 1.0 mg/kg and NE-100 at a dose of 1.0 and 3.0 mg/kg were injected 30 min prior to diazepam or phenazepam. The animals were randomly divided into experimental groups: intact mice (*n* = 15), mice treated with vehicle 1 and vehicle 2 (3 groups; *n* = 15 for each group), mice treated with BD-1047 1.0 mg/kg and vehicle 2 (*n* = 15), mice treated with NE-100 1.0 mg/kg and vehicle 2 (*n* = 15), mice treated with NE-100 3.0 mg/kg and vehicle 2 (*n* = 15), mice treated with vehicle 1 and diazepam 1.0 mg/kg (*n* = 15), mice treated with BD-1047 1.0 mg/kg and diazepam 1.0 mg/kg (*n* = 15), mice treated with NE-100 1.0 mg/kg and diazepam 1.0 mg/kg (*n* = 14), mice treated with NE-100 3.0 mg/kg and diazepam 1.0 mg/kg (*n* = 15), mice treated with vehicle 1 and phenazepam 0.1 mg/kg (*n* = 15), mice treated with BD-1047 1.0 mg/kg and phenazepam 0.1 mg/kg (*n* = 14), and mice treated with NE-100 3.0 mg/kg and phenazepam 0.1 mg/kg (*n* = 14).

(2) Pentylenetetrazole-induced seizures: The Sigma1R agonist PRE-084 and antagonist BD-1047 were dissolved in saline solution for injection (vehicle 1) immediately before administration. Doses of 1, 10, and 20 mg/kg of Sigma1R antagonist BD-1047 and 5 and 20 mg/kg doses of Sigma1R agonist PRE-084 were chosen for the study based on the literature data [142,143,144,145]. Diazepam was dissolved in 20% PEG 400 solution [136,137,138] (vehicle 2) immediately before administration. Administration of 20% PEG 400 solution to experimental animals did not alter seizure thresholds (Figure S6). PTZ was dissolved in saline solution to a concentration of 1% (10 mg/mL) for intravenous infusion. PRE-084, BD-1047, and diazepam were injected intraperitoneally (0.1 mL/10 g body weight), according to the scheme above (Figure 3). PTZ was injected intravenously into the lateral caudal vein according to [146]. 

ICR mice (m = 21–28 g) in the first group were randomly divided into subgroups:

(1) Control group: administration of vehicle 1 and vehicle 2 (*n* = 10);
(2) Three groups: administration of BD-1047 (1, 10, and 20 mg/kg) and vehicle 2 (*n* = 9–11);
(3) Three groups: administration of BD-1047 (1, 10, and 20 mg/kg) and diazepam 1 mg/kg (*n* = 5–8);
(4) Administration of vehicle 1 and diazepam 1 mg/kg (*n* = 8).

ICR mice (m = 24–31 g) in the second group were randomly divided into subgroups:

(1) Control group: administration of vehicle 1 and vehicle 2 (*n* = 12);
(2) Two groups: administration of PRE-084 (5 and 20 mg/kg) and vehicle 2 (*n* = 10–12);
(3) Two groups: administration of PRE-084 (1, 10, and 20 mg/kg) and diazepam 1 mg/kg (*n* = 9–10);
(4) Administration of vehicle 1 and diazepam 1 mg/kg (*n* = 9).

(3) Barbiturate-induced sleep: The Sigma1R agonist PRE-084 and antagonist BD-1047 were dissolved in saline solution for injection immediately before administration. Diazepam was dissolved in 20% PEG 400 solution [136,137,138]. Pentobarbital sodium salt (pentobarbital) was dissolved in saline solution [147]. The studied substances were injected intraperitoneally into mice at the rate of 0.1 mL per 10 g of animal weight. All animals were injected with pentobarbital in the dose of 50 mg/kg 60 min after the first injection of the studied drugs (Figure 6). Immediately after pentobarbital injection, the mice were placed in individual transparent Plexiglas boxes with openings for ventilation.

ICR mice were randomly allocated into six groups:

(1) Control group: administration of saline (*n* = 10);
(2) Two groups: administration of BD-1047 (1 and 10 mg/kg), (*n* = 10);
(3) Two groups, administration of PRE-084 (1 and 5 mg/kg), (*n* = 9–10);
(4) Administration of diazepam 1 mg/kg (*n* = 10).

#### 4.4.2. Elevated Plus Maze Test

The EPM (RPC OpenScience Ltd., Moscow region, Russia) was elevated 40 cm above the floor and illuminated by dim diffused light. The length and width of the arms were 30 cm and 5 cm, respectively; the central area was formed by a 5 × 5 cm square. The wall height of the closed arms was 15 cm. The animals were kept in individual Plexiglass containers after injection on the day of the experiment. For an EPM test, mice were removed from their containers and placed in the central region of the test with their head toward an open arm. The test lasted for 5 min. For each animal, the time in the open arms (T open), time in the center (T center), time in the closed arms (T closed), number of entries into the open arms (N open), number of entries into the closed arms (N closed), and number of visits to the center (N center) were recorded. The total number of test area visits (N total) was calculated as

N total = N open + N center + N closed.(1)

Percentages of open-arm visits and time spent in the open arms were calculated as

%N open = 100 × N open/(N open + N closed),(2)

%T open = 100 × T open/(T open + T closed)(3)

accordingly, abiding by recommendations [148].

#### 4.4.3. Pentylenetetrazole-Induced Seizures

Injection of PTZ into the lateral caudal vein of experimental mice was performed according to previously described methods with some modifications [56,149,150,151,152]. The animals were kept in transparent Plexiglas boxes with holes for ventilation and tail. During the intravenous infusion, the tail was kept outside the box to access the lateral caudal vein, so the animal could move freely in the box without strain on the catheterized tail. PTZ at a 10 mg/mL concentration was injected at a constant rate of 6 μL/s, set on an MD-1020-K BASi Bee Hive Controller 240 V/50 Hz (BASi Corporate Headquarters, West Lafayette, IN, USA) connected to an MD-1001 BASi Bee Baby Bee syringe drive (BASi Corporate Headquarters, West Lafayette, USA). A 27G needle with an attached infusion cannula was injected intravenously into the lateral caudal vein [146]. The insertion site was preheated with an infrared lamp according to the recommendations [56,149,150,151,152]. Accuracy of vein penetration and absence of thrombosis was confirmed by the presence of blood in the catheter. The minimum dose required to induce a seizure was considered the seizure threshold. The infusion was stopped when a generalized tonic seizure was observed. Animal behavior was recorded via video camera and evaluated according to previously described criteria [153]. In this experiment, clonic jerks, generalized clonic seizures, and generalized tonic seizures were recorded.

#### 4.4.4. Barbiturate-Induced Sleep

The test was performed according to previously described methods with some modifications [154,155,156,157,158]. After intraperitoneal infusions, the animals were kept in a transparent Plexiglass box with ventilation holes and sawdust bedding. The animals could move freely without restrictions. Injection of test substances was performed according to the scheme shown above (Figure 6). After intraperitoneal injection of pentobarbital (50 mg/kg), animal behavior was recorded on video. Falling asleep time in seconds was recorded by loss of the righting reflex, sleeping time in seconds was recorded from the moment of falling asleep to the moment of recovery of spontaneous righting reflex.

#### 4.4.5. Statistical Analysis

To evaluate the experimental data distribution, D’Agostino–Pearson, Shapiro–Wilk and Kolmogorov–Smirnov tests were used. Statistical significance was calculated using one-way ANOVA (Sidak or Dunnet post hoc tests) or the Kruskal–Wallis test (Dunn’s post hoc test). The data are presented as a mean with standard deviation (mean ± S.D.), mean with standard error of the mean (mean ± S.E.M.), or median with interquartile range (Mdn (q25–75)). A value of *p* < 0.05 was considered to be statistically significant. Statistical analysis and visualization were performed using GraphPad Prism software version 8.0.1 for Windows (GraphPad, La Jolla, CA, USA, www.graphpad.com (accessed on 28 May 2023)).

### 4.5. In Vitro Radioligand Binding Assay

#### 4.5.1. Membrane Preparation 

BALB/c mice (*n* = 2) were euthanized by cervical dislocation followed by decapitation. The brains (forebrain, intermediate, and midbrain) of each mouse were extracted and homogenized separately in 20 mL of TRIS-HCl buffer solution (pH = 7.4, T = 4 °C) using the Utra-Turrax T25 dispersant (Janke&Kunkel, IKA-Labortechnik, Staufen, Germany) at 24,000 rpm. The obtained suspension was centrifuged at 54,000× *g* for 25 min in an Optima L 70K centrifuge (Beckman Coulter, Brea, CA, USA) at 40C. The resulting precipitate was resuspended in 20 mL of TRIS-HCl buffer solution (pH = 7.4, T = 4 °C) and centrifuged again under same conditions. The resulting precipitate was resuspended in 20 mL of TRIS-HCl buffer solution (pH = 7.4, T = 4 °C). 

#### 4.5.2. Radioligand Binding Procedure

To determine total [N-methyl-^3^H] flunitrazepam binding, 50 μL of 10 nM [N-methyl-^3^H] flunitrazepam solution and 200 μL of cold buffer were added to 250 μL of membrane fraction homogenate. To determine specific binding, 50 μL of 10 nM [N-methyl-^3^H] flunitrazepam solution, 50 μL of diazepam or Sigma1R antagonist BD-1047 or NE-100 solution, and 150 μL of cold buffer were added to 250 μL of membrane fraction homogenate. Incubation was performed for 30 min at T = 4 °C. Radioligand binding was stopped by the addition of 2 mL of ice-cold buffer followed by rapid filtration through GF/B glass fiber filters with subsequent double rinsing with ice-cold buffer to reach a total volume of 8 mL. The radioactivity of the samples was measured on a Tri Carb 2900TR liquid scintillation counter (PerkinElmer, Waltham, MA, USA). The total [N-methyl-^3^H] flunitrazepam binding values obtained were taken as 100%. Specific [N-methyl-^3^H] flunitrazepam binding was calculated as the difference between total and nonspecific binding determined in the presence of diazepam (10 μM). Graphical representation of the data and calculation of the IC50 parameter were performed using GraphPad Prism software version 8.0.1 for Windows (GraphPad, La Jolla, CA, USA, www.graphpad.com (accessed on 28 May 2023)).

## 5. Conclusions

The study shows for the first time the multidirectional effects of compounds with Sigma1R antagonist and agonist properties on GABA_A_Rs-dependent in vivo effects. Sigma1R antagonists inhibited anxiolytic-like, anticonvulsant, and hypnotic effects of GABA_A_Rs PAMs, whereas Sigma1R agonist enhanced their anticonvulsant and hypnotic effects. The obtained results may be a consequence of the ligand influence on the functional activity of Sigma1R, including chaperone interactions with receptors, enzymes and ion channels, regulation of BiP-dependent signaling, and cell membranes remodeling, which requires further studies.

## Data Availability

All data are presented within the manuscript and Supplementary Materials or are available on request from the corresponding authors.

## Supplementary Materials

The following supporting information can be downloaded at: https://www.mdpi.com/article/10.3390/ijms24119580/s1.

## Abbreviations

(+)-SKF 10.047	(1S,9S,13S)-1,13-dimethyl-10-prop-2-enyl-10-azatricyclo [7.3.1.02,7]trideca-2(7),3,5-trien-4-ol
BD-1047	N’-[2-(3,4-dichlorophenyl)ethyl]-N,N,N’-trimethylethane-1,2-diamine
BiP	Endoplasmic reticulum chaperone BiP; GRP78
CB_1_R	Cannabinoid receptors type 1
E1R	(4R,5S)-2-(5-methyl-2-oxo-4-phenyl-pyrrolidin-1-yl)-acetamide
EPM	Elevated plus maze test
ER	Endoplasmic reticulum
GABA	γ-aminobutyric acid
GABA_A_R	GABA_A_ receptor
GluN	NMDA Receptor Subunit
IRE1	Serine/threonine-protein kinase/endoribonuclease IRE1
MT_1_ receptor	Melatonin receptor, type 1
MT_3_ receptor	Melatonin receptor, type 3
NE-100	N-[2-[4-methoxy-3-(2-phenylethoxy)phenyl]ethyl]-N-propylpropan-1-amine
NMDARs	The N-methyl-D-aspartate receptors
NQO2	Ribosyldihydronicotinamide dehydrogenase [quinone]; NRH: quinone reductase 2; QR2
OF	Open-field test
PAMs	Positive allosteric modulators
PKC	Protein kinase C
PRE-084	2-morpholin-4-ylethyl 1-phenylcyclohexane-1-carboxylate
PTZ	Pentylenetetrazole
*Scn1a*	Rodent sodium channel protein type 1 subunit alpha gene
*scn1a*	Zebrafish sodium channel protein type 1 subunit alpha gene
Sigma1R	Sigma nonopioid intracellular receptor 1; Sigma1R chaperon
*Sigmar1*	Rodent Sigma1R gene
SKF83959	9-chloro-3-methyl-5-(3-methylphenyl)-1,2,4,5-tetrahydro-3-benzazepine-7,8-diol;hydrobromide
SOMCL-668	3-methyl-1-phenyl-2,3,4,5-tetrahydro-1H-benzo[d]azepin-7-ol
