# Supplementary material for: Pharmacological Analysis of GABAA Receptor and Sigma1R Chaperone Interaction: Research Report I―Investigation of the Anxiolytic, Anticonvulsant and Hypnotic Effects of Allosteric GABAA Receptors’ Ligands
Source: Int J Mol Sci. 2023 May 31;24(11):9580. doi: 10.3390/ijms24119580 (PMC10253922; doi:10.3390/ijms24119580)
Supplement: Supplementary file 1 [file ijms-24-09580-s001.zip › ijms-2398506-supplementary.pdf]

Supplementary Table S1. The influence of Sigma1R antagonists BD-1047 and NE-100 on mice behavior in the elevated plus-maze test.

**Experimental groups**	**Number of entries into open arms (N open)**	**Number of entries into open arms, % (%N open)**	**Time spent in open arms, s (T open)**	**Time spent in open arms, % (%T open)**	**Number of entries into closed arms (N closed)**	**Number of total entries (N total)**
Intact<br>*n*=15	0.0 (0.0-0.0)	0.0 (0.0-0.0)	0.0 (0.0-0.0)	0.0 (0.0-0.0)	5.27 ± 2.34	10.80 ± 4,69
Veh1+ Veh2<br>*n*=15	0.0 (0.0-1.0)	0.0 (0.0-14.0)	0.0 (0.0-3.0)	0.0 (0.0-1.2)<br>* *p*=0.048	5.07 ± 2.25	10.80 ± 5,52
BD-1047 1.0+ Veh2<br>*n*=15	0.0 (0.0-1.0)	0.0 (0.0-10.0)	0.0 (0.0-2.0)	0.0 (0.0-0.8)	6.67 ± 2.74	14.93 ± 5.97
NE-100 1.0+ Veh2<br>*n*=15	0.0 (0.0-1.0)	0.0 (0.0-10.0)	0.0 (0.0-6.0)	0.0 (0.0-2.6)	5.13 ± 2.13	11.47 ± 4.84
NE-100 3.0+ Veh2<br>*n*=15	0.0 (0.0-1.0)	0.0 (0.0-17.0)	0.0 (0.0-4.0)	0.0 (0.0-1.8)	6.67 ± 2.50	14.80 ± 4.90

Data are presented as median (q25 - q75) for experimental groups N open, %N open, T open, %T open and mean ± S.D for experimental groups N closed, N total. n – the number of animals in the experimental group. Experimental groups: intact BALB/c mice (Intact), vehicle 1 + vehicle 2 (Veh1+ Veh2), BD-1047 1.0 mg/kg + vehicle 2 (BD-1047 1.0+ Veh2), NE-100 1.0 mg/kg + vehicle 2 (NE-100 1.0+ Veh2), NE-100 3.0 mg/kg + vehicle 2 (NE-100 3.0+ Veh2). Kruskal–Wallis test, Dunn's multiple comparison test: * - statistical significance vs. Intact.

Supplementary Table S2. The influence of Sigma1R antagonists BD-1047 and NE-100 on the anxiolytic effect of diazepam in the elevated plus-maze test.

Experimental groups	Number of entries into open arms (N open)	Number of entries into open arms, % (%N open)	Time spent in open arms, s (T open)	Time spent in open arms, % (%T open)	Number of entries into closed arms (N closed)	Number of total entries (N total)
Veh1+ Veh2<br>*n*=15	0.0 (0.0-1.0)	0.0 (0.0-13.0)	0.0 (0.0-3.0)	0.0 (0.0-0.8)	5.73 ± 2.63	12.60 ± 5.71
Veh1+D 1.0<br>*n*=15	6.0 (5.0-9.0)<br>* p<0.001	46.0 (33.0-72.0)<br>* p<0.001	93.0 (77.0-201.0)<br>* p<0.001	39.0 (32.0-85.0)<br>* p<0.001	6.73 ± 4.22	28.0 ± 9.61<br>* p<0.001
BD-1047 1.0+D 1.0<br>*n*=15	4.0 (2.0-5.0)	25.0 (19.0-36.0)<br># p=0.025	26.0 (19.0-37.0)<br># p=0.0016	10.0 (7.2-15.0)<br># p=0.002	10.60 ± 3.68<br># p=0.0054	29.40 ± 9.87
NE-100 1.0+D 1.0<br>*n*=14	6.0 (3.75-8.0)	50.0 (32.25-53.75)	79.5 (30.25-190.3)	34.95 (11.18-69.78)	7.36 ± 2.24	26.43 ± 6.27
NE-100 3.0+D 1.0<br>*n*=15	4.0 (3.0-6.0)	33.0 (20.0-39.0)	33.0 (14.0-59.0)<br># p=0.011	13.0 (5.2-24.0)<br># p=0.011	8.13 ± 2.59	25.27 ± 10.07

Data are presented as median (q25 - q75) for experimental groups N open, %N open, T open, %T open and mean ± S.D for experimental groups N closed, N total. n – the number of animals in the experimental group. Experimental groups: vehicle 1 + vehicle 2 (Veh1+Veh2), vehicle 1 + diazepam 1.0 mg/kg (Veh1+D 1.0), BD-1047 1.0 mg/kg + diazepam 1.0 mg/kg (BD-1047 1.0+D 1.0), NE-100 1.0 mg/kg + diazepam 1.0 mg/kg (NE-100 1.0+D 1.0), NE-100 3.0 mg/kg + diazepam 1.0 mg/kg (NE-100 3.0+D 1.0). Kruskal–Wallis test, Dunn's multiple comparison test for experimental groups N open, %N open, T open, %T open, one-way ANOVA and the post hoc Sidak multiple comparisons test for experimental groups N closed, N total: * - statistical significance vs. Veh1+Veh2, # - statistical significance vs. Veh1+D 1.0.

Supplementary Table S3. The influence of Sigma1R antagonists BD-1047 and NE-100 on the anxiolytic effect of phenazepam in the elevated plus-maze test.

Experimental groups	Number of entries into open arms (N open)	Number of entries into open arms, % (%N open)	Time spent in open arms, s (T open)	Time spent in open arms, % (%T open)	Number of entries into closed arms (N closed)	Number of total entries (N total)
Veh1+Veh2 *n*=15	1.0 (0.0-1.0)	8.03 (0.0-17.0)	0.0 (0.0-3.0)	0.0 (0.0-1.1)	5.73 ± 2.60	12.93 ± 5.18
Veh1+Ph 0.1 *n*=15	5.0 (3.0-7.0) * $p<0.001$	47.0 (39.0-57.0) * $p<0.001$	119.0 (77.0-189.0) * $p<0.001$	56.3 (33.6-76.7) * $p<0.001$	5.73 ± 3.31	21.80 ± 9.03 * $p=0.001$
BD-1047 1.0+Ph 0.1 *n*=14	2.0 (1.0-3.0) # $p=0.011$	22.0 (20.0-36.5) # $p=0.016$	25.0 (12.75-40.25) # $p=0.019$	10.1 (4.47-14.93) # $p=0.016$	5.71 ± 2.27	15.43 ± 5.39 # $p=0.031$
NE-100 3.0+ Ph 0.1 *n*=14	1.0 (0.75-2.0) # $p<0.001$	19.0 (9.75-26.0) # $p<0.001$	13.5 (0.75-21.75) # $p<0.001$	5.0 (0.3-8.2) # $p<0.001$	5.57 ± 1.99	13.93 ± 5.28 # $p=0.006$

Data are presented as median (q25 - q75) for experimental groups N open, %N open, T open, %T open and mean ± S.D for experimental groups N closed, N total. n – the number of animals in the experimental group. Experimental groups: vehicle 1 + vehicle 2 (Veh1+ Veh2), vehicle 1 + phenazepam 0.1 mg/kg (Veh1+Ph 0.1), BD-1047 1.0 mg/kg + phenazepam 0.1 mg/kg (BD-1047 1.0+Ph 0.1), NE-100 3.0 mg/kg + phenazepam 0.1 mg/kg (NE-100 3.0+Ph 0.1). Kruskal–Wallis test, Dunn's multiple comparison test for experimental groups N open, %N open, T open, %T open, one-way ANOVA and the post hoc Sidak multiple comparisons test for experimental groups N closed, N total: * - statistical significance vs. Veh1+Veh2, # - statistical significance vs. Veh1+Ph 0.1.

Supplementary Table S4. The influence of Sigma1R agonist PRE-084 on the anxiolytic effect of diazepam in the elevated plus-maze test.

Experimental groups	Number of entries into open arms (N open)	Number of entries into open arms, % (%N open)	Time spent in open arms, s (T open)	Time spent in open arms, % (%T open)	Number of entries into closed arms (N closed)	Number of total entries (N total)
Veh1+Veh2 *n*=8	0.0 (0.0-0.0)	0.0 (0.0-0.0)	0.0 (0.0-0.0)	0.0 (0.0-0.0)	4.63 ± 2.93	9.63 ± 5.98
Veh1+D 1.0 *n*=8	7.0 (6.25-9.75) *p=0.001	56.5 (47.0-59.75) *p<0.001	124.0 (118.5-144.5) *p=0.004	49.05 (42.23-55.25) *p=0.026	6.5 ± 1.77	28.88 ± 5.54 *p<0.001
PRE-084 1.0+D 1.0 *n*=8	7.0 (5.25-10.5) *p=0.002	47.5 (36.5-52.75) *p=0.007	131.0 (120.3-186.0) *p<0.001	62.5 (55.53-67.85) *p<0.001	8.38 ± 3.46 *p=0.042	32.25 ± 11.78 *p<0.001

Data are presented as median (q25-q75) for experimental groups N open, %N open, T open, %T open and mean ± S.D for experimental groups N closed, N total. n – the number of animals in the experimental group. Experimental groups: vehicle 1 + vehicle 2 (Veh1+ Veh2), vehicle 1 + diazepam 1.0 mg/kg (Veh1+D 1.0), PRE-084 1.0 mg/kg + diazepam 1.0 mg/kg (PRE-084 1.0+D 1.0). Kruskal–Wallis test, Dunn's multiple comparison test for experimental groups N open, %N open, T open, %T open, one-way ANOVA and the post hoc Sidak multiple comparisons test for experimental groups N closed, N total: * - statistical significance vs. Veh1+Veh2.

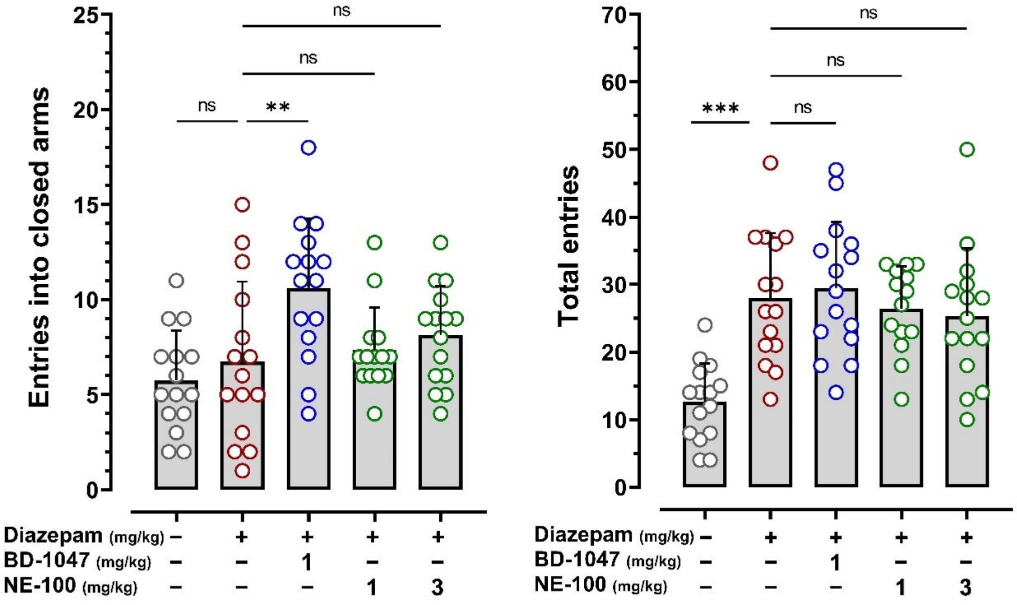

(a) **(b)**

Supplementary Figure S1. Influence of Sigma1R antagonists BD-1047 and NE-100 on the effect of diazepam evaluated by the parameters "entries into closed arm" and "total entries" in the elevated plus-maze test. (**a**) The number of entries into the closed arms (N closed); (**b**) The number of total entries into the open and closed arms (N total). Vehicle 2 and diazepam (1.0 mg/kg) were injected i.p. 30 min prior to the EPM exposition. Vehicle 1, selective Sigma1R antagonists BD-1047 (1.0 mg/kg) and NE-100 (1.0 and 3.0 mg/kg) were injected i.p. 30 min prior diazepam. Data are presented as mean with SD. Statistically significant differences according to the one-way ANOVA and the post hoc Sidak multiple comparisons test: ns - not significant; ** $p < 0.01$; *** $p < 0.001$.

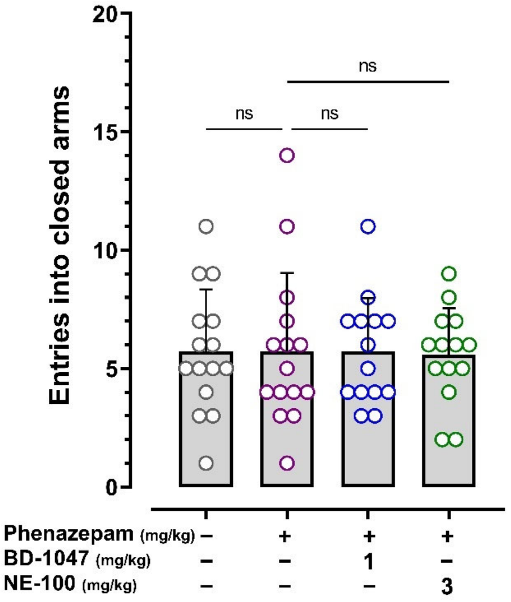

(a)

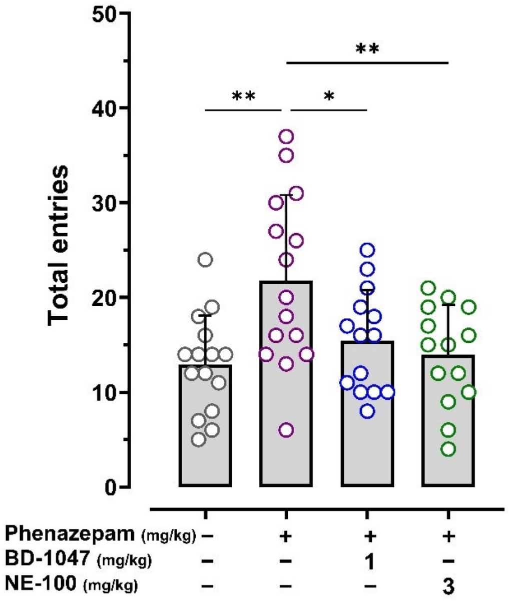

(b)

Supplementary Figure S2. Influence of Sigma1R antagonists BD-1047 and NE-100 on the effect of phenazepam evaluated by the parameters "entries into closed arm" and "total entries" in the elevated plus-maze test. (**a**) The number of entries into the closed arms (N closed); (**b**) The number of total entries into the open and closed arms (N total). Vehicle 2 and phenazepam (0.1 mg/kg) were injected i.p. 30 min prior to the EPM exposition. Vehicle 1, selective Sigma1R antagonists BD-1047 (1.0 mg/kg) and NE-100 (3.0 mg/kg) were injected i.p. 30 min prior phenazepam. Data are presented as mean with SD. Statistically significant differences according to the one-way ANOVA and the post hoc Sidak multiple comparisons test: ns - not significant; * $p < 0.05$; ** $p < 0.01$.

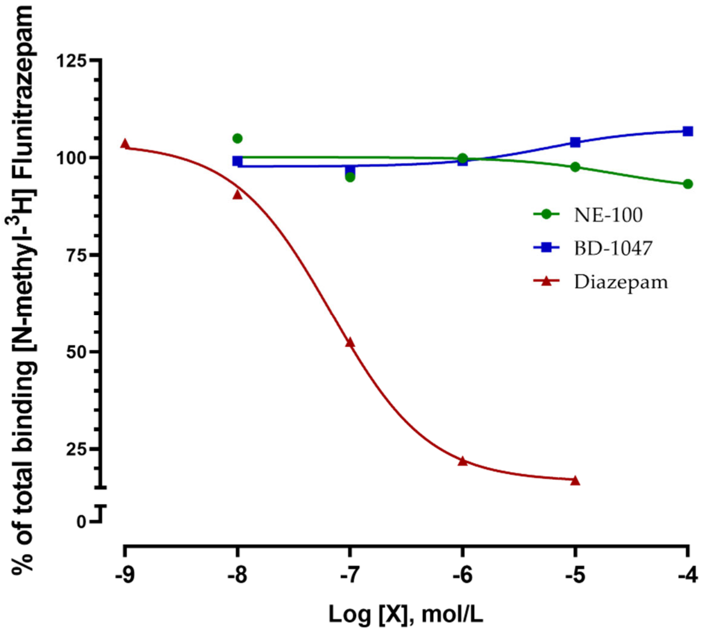

Supplementary Figure S3. Competitive interaction of diazepam and Sigma1R antagonists BD-1047 and NE-100 with [N-methyl-^{3}H] Flunitrazepam. Data were obtained in the brain homogenates of BALB/c mice (*n*=2). IC_{50} diazepam = 68 nM.

Supplementary Table S5. The influence of Sigma1R antagonist BD-1047 on anticonvulsant activity of diazepam in the intravenous pentylenetetrazol infusion test on mice.

Experimental groups	Dose of PTZ to induce clonic jerks, mg/kg<br>Mean ± SEM (min;max)	Dose of PTZ to induce generalized clonic seizure, mg/kg<br>Mean ± SEM (min;max)	Dose of PTZ to induce generalized tonic seizure, mg/kg<br>Mean ± SEM (min;max)
**1% PTZ**<br>*n*=10	34.86 ± 1.65<br>(24;41)	43.52 ± 2.47<br>(34;60)	100.39 ± 5.12<br>(73;123)
**BD-1047 1 mg/kg, 1% PTZ**<br>*n*=11	39.49 ± 1.30<br>(30;45)	46.76 ± 2.11<br>(34;56)	103.47 ± 5.82<br>(70;136)
**BD-1047 10 mg/kg, 1% PTZ**<br>*n*=9	35.50 ± 2.26<br>(29;52)	47.06 ± 4.01<br>(35;77)	102.46 ± 9.59<br>(53;139)
**BD-1047 20 mg/kg, 1% PTZ**<br>*n*=9	33.14 ± 2.60<br>(24;47)	42.86 ± 2.56<br>(32;53)	86.28 ± 10.02<br>(48;128)
**Diaz 1 mg/kg, 1% PTZ**<br>*n*=8	102.46 ± 9.59<br>(55;83) #### p<0.0001	81.32 ± 3.31<br>(68;95) #### p<0.0001	181.09 ± 7.10<br>(146;299) #### p<0.0001
**BD-1047 1 mg/kg, Diaz 1 mg/kg, 1% PTZ**<br>*n*=5	70.65 ± 3.55<br>(60;81)	91.14 ± 5.15<br>(73;103)	157.66 ± 18.51<br>(110;208)
**BD-1047 10 mg/kg, Diaz 1 mg/kg, 1% PTZ**<br>*n*=8	68.31 ± 3.13<br>(56;81)	80.26 ± 2.69<br>(68;90)	149.79 ± 7.64<br>(115;174)
**BD-1047 20 mg/kg, Diaz 1 mg/kg, 1% PTZ**<br>*n*=7	50.37 ± 1.58<br>(42;55) **** p<0.0001	64.39 ± 3.76<br>(55;81) ** p=0.0057	133.54 ± 10.58<br>(99;166) ** p=0.0068

n – the number of animals in the experimental group. Diaz – Diazepam. Statistically significant differences according to the one-way ANOVA and the post hoc Dunnet multiple comparisons test: * - vs. Diaz 1 mg/kg, 1% PTZ; # - vs. 1% PTZ.

Supplementary Table S6. The influence of Sigma1R agonist PRE-084 on anticonvulsant activity of diazepam in the intravenous pentylenetetrazol infusion test on mice.

Experimental groups	Dose of PTZ to induce clonic jerks, mg/kg<br>Mean ± SEM (min;max)	Dose of PTZ to induce generalized clonic seizure, mg/kg<br>Mean ± SEM (min;max)	Dose of PTZ to induce generalized tonic seizure, mg/kg<br>Mean ± SEM (min;max)
**1% PTZ**<br>*n*=12	43.86 ± 1.47<br>(37; 53)	51.9 ± 1.53<br>(43; 63)	67.67 ± 3.42<br>(50; 83)
**PRE–084 5 mg/kg, 1% PTZ**<br>*n*=12	49.36 ± 2.23<br>(40; 61)	57.81 ± 3.11<br>(44; 73)	74.7 ± 6.15<br>(50; 103)
**PRE–084 20 mg/kg, 1% PTZ**<br>*n*=11	43.2 ± 1.33<br>(38; 50)	50.35 ± 2.23<br>(38; 66)	95.53 ± 5.66<br>(73; 127)
**Diaz 1 mg/kg, 1% PTZ**<br>*n*=10	69.25 ± 2.31<br>(55; 78) #### $p<0.0001$	84.46 ± 2.22<br>(77; 94) #### $p<0.0001$	101 ± 5.21<br>(82; 127) ## $p=0.0055$
**PRE–084 5 mg/kg, Diazepam 1 mg/kg, 1% PTZ**<br>*n*=11	85.2 ± 2.93<br>(67; 102) **** $p<0.0001$	101.5 ± 4.36<br>(82; 133) ** $p=0.0038$	130.6 ± 7.89<br>(108; 174) * p=0.0272
**PRE–084 20 mg/kg, Diazepam 1 mg/kg, 1% PTZ**<br>*n*=12	81.4 ± 2.78<br>(68; 97) ** $p=0.002$	91.42 ± 3.91<br>(72; 115)	155.8 ± 11.46<br>(88; 218) **** $p<0.0001$

n – the number of animals in the experimental group. Diaz – Diazepam. Statistically significant differences according to the one-way ANOVA and the post hoc Dunnet multiple comparisons test: * - vs. Diaz 1 mg/kg, 1% PTZ; # - vs. 1% PTZ.

Supplementary Table S7. The influence of Sigma1R antagonist BD-1047 and agonist PRE-084 on mice falling asleep time and sleeping time in the pentobarbital-induced sleep test on mice.

Experimental groups	Falling asleep time, s<br>Mean ± SEM (min;max)	Sleeping time, s<br>Mean ± SEM (min;max)
**Pentobarbital sodium 50 mg/kg**<br>*n*=10	229 ± 5.63<br>(206; 262)	5842.7 ± 277.05<br>(4763; 6870)
**BD-1047 1 mg/kg**<br>**Pentobarbital sodium 50 mg/kg**<br>*n*=10	260 ± 7.33<br>(233; 308)<br>** p=0.0023	4900.5 ± 84.14<br>(4370; 5213)<br>** p=0.0037
**BD-1047 10 mg/kg**<br>**Pentobarbital sodium 50 mg/kg**<br>*n*=10	240.2 ± 4.91<br>(216; 263)	4944.9 ± 171.45<br>(3935; 5625)<br>** p=0.0056
**PRE-084 1 mg/kg**<br>**Pentobarbital sodium 50 mg/kg**<br>*n*=10	222.8 ± 17.59<br>(150; 290)	11078.56 ± 1632.8<br>(4680; 19160)<br>*** p=0.0008
**PRE-084 5 mg/kg**<br>**Pentobarbital sodium 50 mg/kg**<br>*n*=10	190.1 ± 8.98<br>(150; 230)<br>* p=0.0473	12246 ± 665.69<br>(8980; 15579)<br>**** p<0.0001
**Diazepam 1 mg/kg**<br>**Pentobarbital sodium 50 mg/kg**<br>*n*=10	244 ± 8.46<br>(210; 285)	11336.5 ± 641.45<br>(8730; 14580)<br>*** p=0.0003

n – the number of animals in the experimental group. Statistically significant differences according to the one-way ANOVA and the post hoc Dunnet multiple comparisons test: * - statistical significance vs. Pentobarbital sodium 50 mg/kg. * $p < 0.05$; ** $p < 0.01$; *** $p < 0.001$; **** $p < 0,0001$.

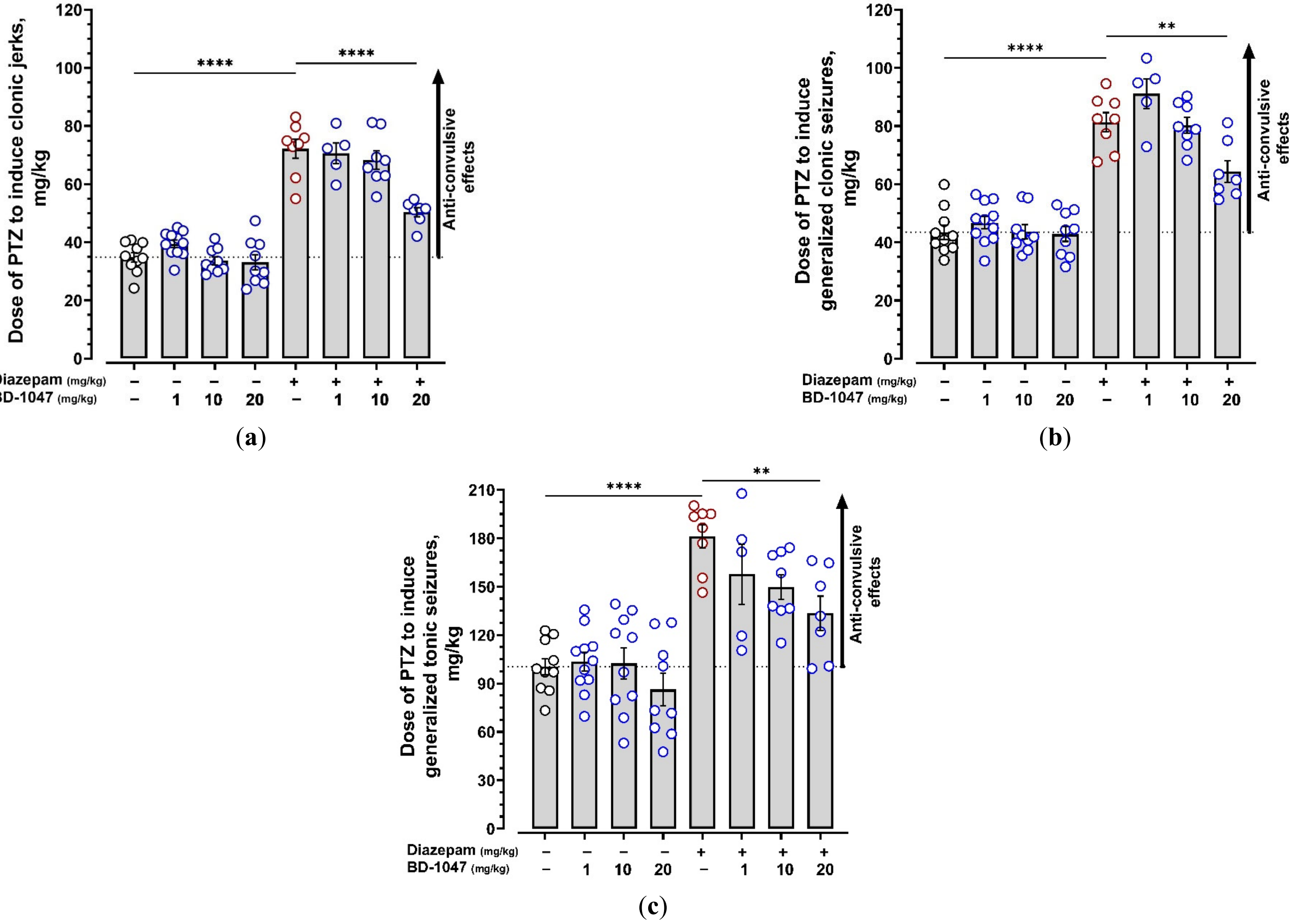

Supplementary Figure S4. The influence of Sigma1R antagonist BD-1047 on anticonvulsant activity of diazepam in the intravenous pentylenetetrazol infusion test on mice. (**a**) Clonic jerks; (**b**) Generalized clonic seizure; (**c**) Generalized tonic seizure. Data are presented as mean ± S.E.M. Statistically significant differences according to the one-way ANOVA and the post hoc Dunnet multiple comparisons test: ** $p < 0.01$; ****$p < 0.0001$.

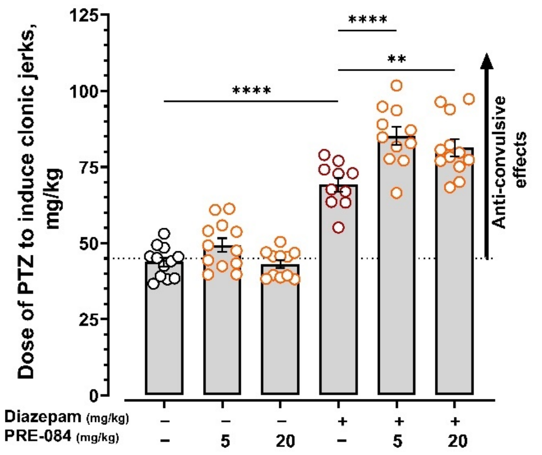

(a)

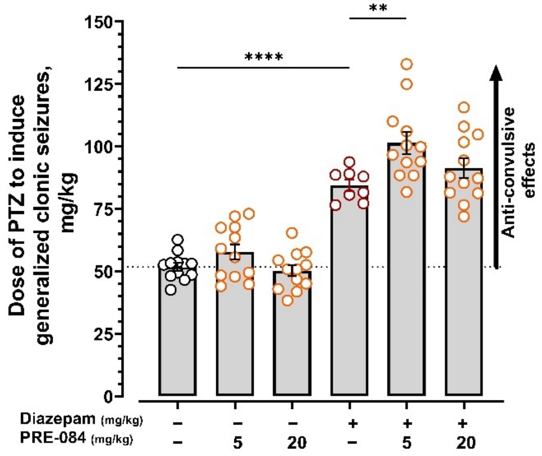

(b)

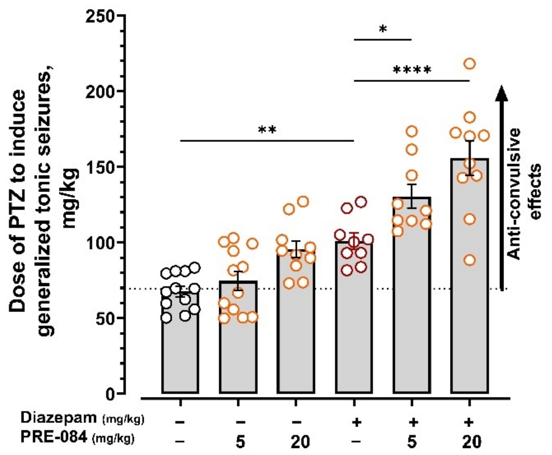

(c)

Supplementary Figure S5. The influence of Sigma1R agonist PRE-084 on anticonvulsant activity of diazepam in the intravenous pentylenetetrazol infusion test on mice. (**a**) Clonic jerks; (**b**) Generalized clonic seizure; (**c**) Generalized tonic seizure. Data are presented as mean ± S.E.M. Statistically significant differences according to the one-way ANOVA and the post hoc Dunnet multiple comparisons test: * $p < 0{,}05$; ** $p < 0.01$; **** $p < 0.0001$.

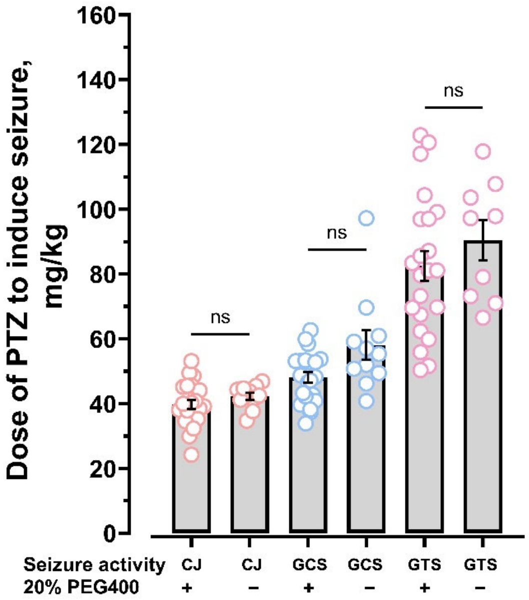

Supplementary Figure S6. The influence of 20% PEG 400 on convulsant activity of PTZ in the intravenous pentylenetetrazol infusion test on mice. CJ: clonic jerks, GCS: generalized clonic seizure, GTS: generalized tonic seizure. Data are presented as mean ± S.E.M. Statistically significant differences according to the one-way ANOVA and the post hoc Dunnet multiple comparisons test: ns - not significant
